# Analysis on the characteristics of spatio-temporal evolution and aggregation trend of early COVID-19 in mainland China

**DOI:** 10.1038/s41598-022-08403-w

**Published:** 2022-03-14

**Authors:** Shengxian Bi, Siyu Bie, Xijian Hu, Huiguo Zhang

**Affiliations:** Department of Mathematics and Systems Science, Xinjiang University, Urumqi, 830046 China

**Keywords:** Influenza virus, Statistics

## Abstract

To analyze the spatio-temporal aggregation of COVID-19 in mainland China within 20 days after the closure of Wuhan city, and provide a theoretical basis for formulating scientific prevention measures in similar major public health events in the future. Draw a distribution map of the cumulative number of COVID-19 by inverse distance weighted interpolation; analyze the spatio-temporal characteristics of the daily number of COVID-19 in mainland China by spatio-temporal autocorrelation analysis; use the spatio-temporal scanning statistics to detect the spatio-temporal clustering area of the daily number of new diagnosed cases. The cumulative number of diagnosed cases obeyed the characteristics of geographical proximity and network proximity to Hubei. Hubei and its neighboring provinces were most affected, and the impact in the eastern China was more dramatic than the impact in the western; the global spatio-temporal Moran’s I index showed an overall downward trend. Since the 10th day of the closure of Wuhan, the epidemic in China had been under effective control, and more provinces had shifted into low-incidence areas. The number of new diagnosed cases had gradually decreased, showing a random distribution in time and space (P< 0.1), and no clusters were formed. Conclusion: the spread of COVID-19 had obvious spatial-temporal aggregation. China’s experience shows that isolation city strategy can greatly contain the spread of the COVID-19 epidemic.

## Introduction

COVID-19 is an infectious disease whose main symptoms were breathing, coughing and sneezing. The median incubation period is 3 days and the longest can be up to 24 days^1^. The COVID-19 had spread to the world from the beginning of 2020. By December 29, 2021, the cumulative number of diagnosed cases worldwide has exceeded 280 million, and the cumulative number of deaths has exceeded 5 million^2^. It took only 2 or 3 months from the outbreak of COVID-19 to containment in mainland China. The arduous path of epidemic prevention in mainland China has provided valuable experience for all countries in the world.

Various work^3–9^ has analyzed the pathological and epidemiological characteristics of COVID-19. Literature^10,11^ have used infectious disease dynamic models (SEIR, LES) to predict the spread of COVID-19, which show that COVID-19 infection is spatially dependent and can spread to nearby areas. Literature^12,13^ have paid attention to the risk of COVID-19 spreading in urban and rural areas, and find that COVID-19 epidemic is composed of sub-epidemics with different temporal dynamics and spatial patterns. Some scholars have studied the regional transmission mechanism of COVID-19 through models such as GWR and metric geometry^14,15^, and find that the pattern of spatial differentiation of COVID-19 is a transitional pattern of parallel bands from east to west, and also an epitaxial radiation pattern centered in the Wuhan 1 + 8 urban circle. Some scholars also use geographic information systems and spatial autocorrelation analysis to uncover the geographic distribution characteristics of COVID-19. For example: Su^16^ uses spatial autocorrelation analysis at multiple time points to study the spatial aggregation of the cumulative number of confirmed cases in various regions of China. Liu^17^ assesses the spread of the epidemic in Henan province through spatial autocorrelation analysis and relative risk coefficients. Jian^18^ describes the spatial pattern and distribution characteristics of the epidemic situation in Henan province from the perspective of planning. These studies have achieved important results, explaining the epidemic mechanism of COVID-19 in time and space.

COVID-19 was detected and spread in Wuhan, Hubei province in January 2020. Because of the huge population of Wuhan and the proximity of the Chinese Lunar New Year, COVID-19 quickly spread to all provinces in mainland China. Among them, Hubei province and its surrounding provinces are the most affected. The closure strategy adopted by Wuhan has significantly contained the risk of epidemics caused by COVID-19. This study focuses on the spatio-temporal distribution of COVID-19 in mainland China when the epidemic just broke out. At present, there are relatively few studies on the correlation of the epidemic in the two dimensions of time and space during this time period in mainland China. This study uses spatio-temporal autocorrelation analysis and spatio-temporal scanning statistical methods to detect the spatio-temporal aggregation of COVID-19 and provide a theoretical basis for scientifically formulating epidemic prevention measures.

## Results

### Analysis of inverse distance weighted interpolation

**Figure 1:**
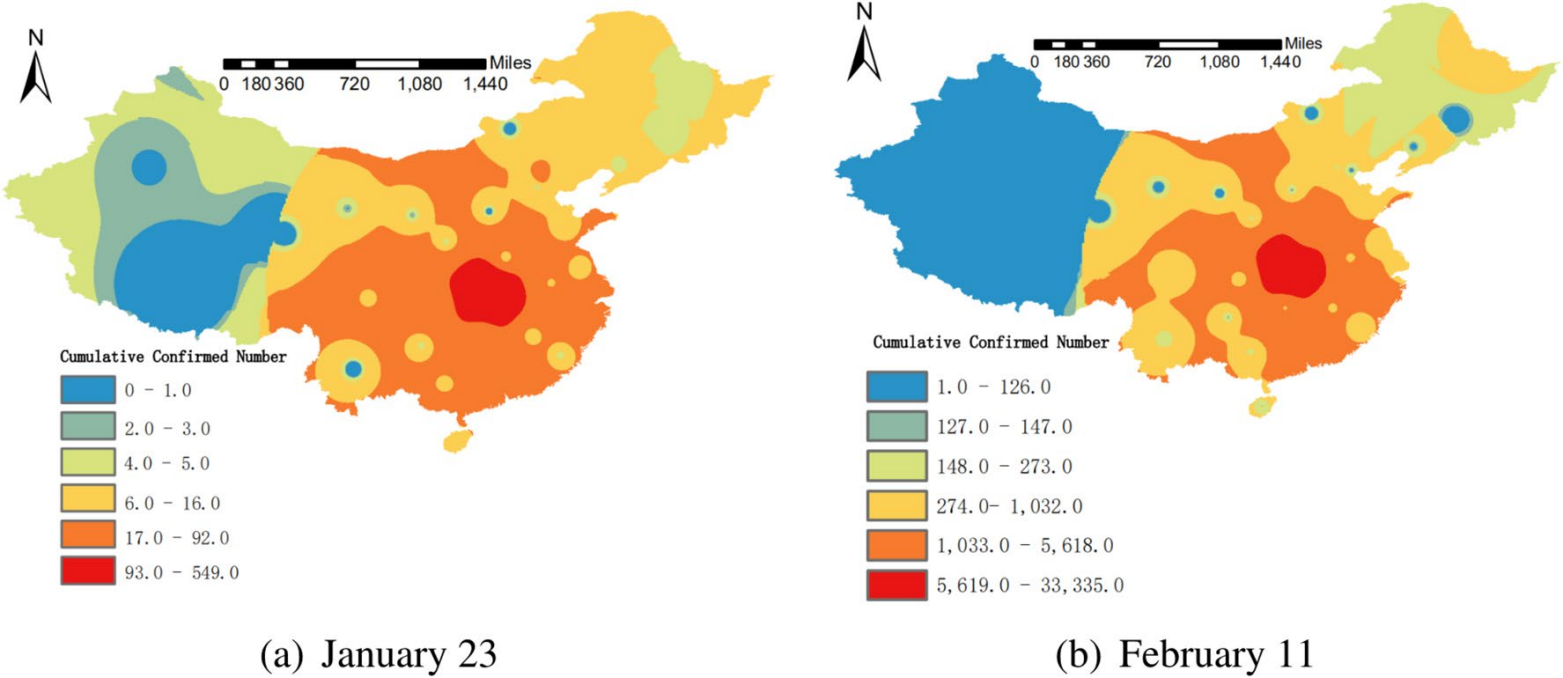
Time-series diagram of spatio-temporal Moran’s I index changes. Maps constructed using ArcGIS 10.2 (https://s2.loli.net/2022/01/11/MwZ281HAeBbN3uP.png).

The spatial distribution characteristics of the cumulative number of diagnosed cases with COVID-19 in mainland China on January 23 and February 11 are analyzed by inverse distance weighted interpolation, and the distribution map is drawn. The results are shown in Fig. 1. The spatial distribution map showed that the epidemic situation had basically spread to the whole country on January 23. Compared with February 11, most of the high incidence areas (hot spots) and low incidence areas (cold spots) of the epidemic were the same, and there was no larger-scale spread, indicating that the epidemic has been better controlled during this period. On the whole, the effect to Tibet, Yunnan, Northwest China, and Northeast China were less dramatic than Hubei and its neighboring regions. The cumulative number of diagnosed cases obeyed the characteristics of Hubei’s geographic proximity and network proximity, which was consistent with relevant research conclusions^17^.

### Spatio-temporal autocorrelation analysis

#### Global spatio-temporal autocorrelation analysis

**Figure 2:**
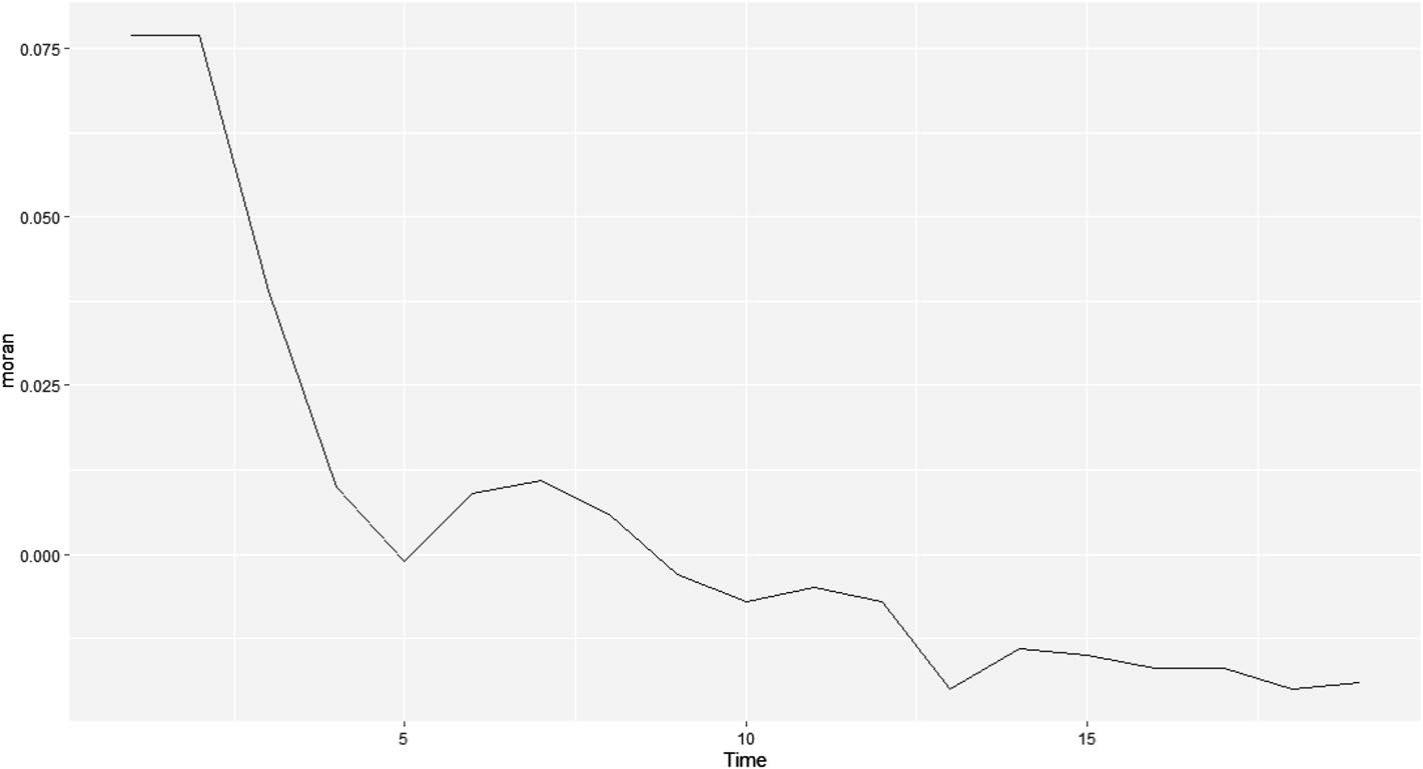
Time-series diagram of spatio-temporal Moran’s I index changes.

The global spatio-temporal Moran’s I index can be used to describe the degree of spatio-temporal autocorrelation at two different moments in the study area. In Table 1 and Fig. 2, we display the global spatio-temporal autocorrelation analysis of the number of new diagnosed cases from January 23 to February 11, computed in (2), with the lag order k=1. From January 23 to January 31, the daily global spatio-temporal Moran’s I index was almost positive with the p-value less than 0.1, and showed a downward trend, with the most obvious decline in the first 5 days. It showed that with the onset of the incubation period cases with a history of contact in Hubei, the epidemic was increasing rapidly during this period, and there was a spatio-temporal aggregation, but it was quickly under control. From February 1 to February 4, the daily global spatio-temporal Moran’s I index was negative, and the value was small and close to 0. The significance test indicated that the daily number of new diagnosed cases passed the peak and began to show a weak downward trend, and there was still spatio-temporal aggregation. From February 5 to February 11, the global spatio-temporal Moran’s I index continued to be negative, with a relatively large value. It failed the significance test, indicating that the epidemic began to get under control, and the daily number of new diagnosed cases showed a significant downward trend, which was randomly distributed in time and space.

**Table 1:** Results of spatio-temporal autocorrelation analysis of the new diagnosed cases of COVID-19 in Mainland China.

T-K time	T time	Moran’s I index	*Z*-value	*P*-value	Status
January 23	January 24	0.077	2.03	0.040	Clustering
January 24	January 25	0.077	2.61	0.008	Clustering
January 25	January 26	0.039	1.95	0.040	Clustering
January 26	January 27	0.010	1.68	0.030	Clustering
January 27	January 28	− 0.001	1.28	0.090	Clustering
January 28	January 29	0.009	1.37	0.090	Clustering
January 29	January 30	0.011	1.41	0.080	Clustering
January 30	January 31	0.006	1.43	0.080	Clustering
January 31	February 1	− 0.003	1.37	0.070	Clustering
February 1	February 2	− 0.007	1.36	0.070	Clustering
February 2	February 3	− 0.005	1.45	0.060	Clustering
February 3	February 4	− 0.007	1.39	0.060	Clustering
February 4	February 5	− 0.020	1.04	0.120	Random distribution
February 5	February 6	− 0.014	1.00	0.140	Random distribution
February 6	February 7	− 0.015	1.04	0.130	Random distribution
February 7	February 8	− 0.017	1.03	0.140	Random distribution
February 8	February 9	− 0.017	0.97	0.140	Random distribution
February 9	February 10	− 0.020	0.81	0.200	Random distribution
February 10	February 11	− 0.019	0.88	0.170	Random distribution

#### Local spatio-temporal autocorrelation analysis

**Figure 3:**
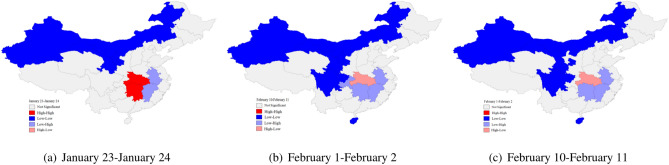
The spatio-temporal aggregation of the new diagnosed cases of COVID-19. Maps constructed using GeoDa 1.12.1 (https://s2.loli.net/2022/01/11/5CZKuTaztYeD8wN.png).

Local spatio-temporal autocorrelation analysis is used to identify the correlation patterns of different spatio-temporal locations, and local spatio-temporal non-stationarity can be observed. In Fig. 3, we display the local spatio-temporal autocorrelation analysis, computed in (4), with the lag order k=1. Figure 3 shows the local spatio-temporal aggregation of new diagnosed cases at three different time points after the city was closed, and all of them passed the significance test.

High-high clustering pattern means that the neighboring areas of the area with a large number of new diagnosed cases at the current moment will have more new diagnosed cases at the next moment. Low-low clustering pattern means that the neighboring areas of the area with few new diagnosed cases at the current moment will also have fewer new diagnosed cases at the next moment. High-low clustering pattern means that the neighboring areas of the area with a large number of new diagnosed cases at the current moment will have fewer new diagnosed cases at the next moment. Low-high clustering pattern means that the number of new diagnosed cases will increase in the neighbouring areas of the area with few new diagnosed cases at the current moment^24^.

The high-high clustering pattern was mainly concentrated in Hubei and Hunan in the early epidemic, and their neighboring provinces such as Anhui and Jiangxi were more affected. With the deepening of national epidemic prevention work, the number of new diagnosed cases in Hubei began to decrease and transformed into a high-low cluster mode on February 1. The daily number of new diagnosed cases in Hunan, Chongqing, Anhui, Jiangxi and other regions began to decline gradually, which was consistent with the results of global spatio-temporal autocorrelation analysis. The low-low clustering pattern mainly focused on regions from Xinjiang, Inner Mongolia, Gansu, and Ningxia in the early epidemic, and successively joining some provinces such as Sichuan and Hainan, suggesting that the fundamentals of the national epidemic have gradually improved during this period.

### Spatio-temporal scanning statistics analysis

**Figure 4:**
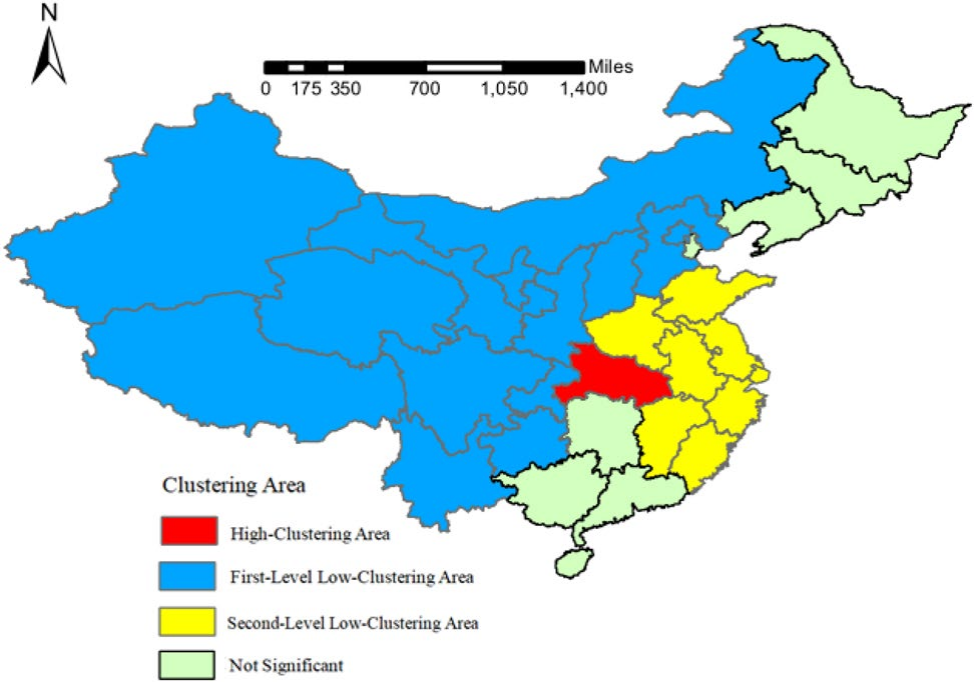
The spatio-temporal aggregation of the new diagnosed cases of COVID-19. Maps constructed using ArcGIS 10.2 (https://s2.loli.net/2022/01/11/fA85DxBvaUEGbmK.png).

The results of the spatio-temporal scanning statistics analysis are shown in Table 2 and Fig. 4, computed by Eq. (5). The results show that there are three spatio-temporal clusters for the number of new diagnosed cases in the country from January 23 to February 11. If the relative risk index (RR) is more than 1, the cluster is a high-clustering area, otherwise, the cluster is a low-clustering area. The p-values of the significance test results are all less than 0.001, which are statistically significant. The high-clustering area was Hubei, the clustering time was from February 1 to February 10, and the relative risk index was 76.84. There were two low-clustering areas. The first cluster included Xinjiang, Qinghai, Ningxia, Gansu, Shaanxi, Tibet, Sichuan, Guizhou, Chongqing, Yunnan, Beijing, Shanxi, Hebei and Inner Mongolia, with a relative risk index of 0.095. The second cluster included Shanghai, Jiangsu, Zhejiang, Anhui, Fujian, Jiangxi, Shandong and Henan, with a relative risk index of 0.42, and the clustering time was from January 23 to February 1. The results of the spatio-temporal scanning show that the effect to neighboring provinces of Hubei and developed coastal areas were more dramatic than the northwest China, southwest China and northern China in the first 10 days of early epidemic. From the 10th day to the 20th day, the number of new diagnosed cases in other regions of the country except Hubei was randomly distributed, and there was no clustering area in time and space.

**Table 2:** The spatio-temporal scanning aggregation results of the new diagnosed cases of COVID-19, January 23–February 11.

Clustering Pattern	Clustering Area	Clustering Time	RR	LLR	p-value
high-clustering	Hubei	February 1–February 10	76.84	71,476.71	< 0.001
first-level low-clustering	Xinjiang, Qinghai, Ningxia, Gansu, Shaanxi, Tibet, Sichuan, Guizhou, Chongqing, Yunnan, Beijing, Shanxi, Hebei, Inner Mongolia	January 23–February 1	0.095	7292.43	< 0.001
Second-level low-clustering	Shanghai, Jiangsu, Zhejiang, Anhui, Fujian, Jiangxi, Shandong, Henan	January 23–February 1	0.42	1191.71	< 0.001

## Discussion

Based on the spatio-temporal analysis of the cumulative number of COVID-19 confirmed cases and the number of new diagnosed cases in various regions of China from January 23 to February 11, this article introduces a time dimension on the basis of traditional spatial statistics, and systematically reflects the trend of the spread of COVID-19 and spatio-temporal clustering area. Results show that various regions of China were affected by the epidemic on January 23, but it was quickly prevented and controlled, and it did not spread to a larger area. The effect to Hubei and its neighboring provinces was most dramatic, and the effect to the eastern region is less dramatic than the western region. Since the 10th day of Wuhan’s isolation, the epidemic in China had been effectively controlled, and more provinces had changed into low-incidence area, and the daily number of new diagnosed cases had gradually decreased. It appeared to be randomly distributed in time and space, and no clustering areas were formed. The conclusion shows that the active epidemic prevention measures adopted by China had effectively contained the further spread of the epidemic and minimized the possibility of the spread of the virus. In the future, we should act quickly and do our utmost to avoid the spread of the epidemic in similar major public health incidents. At the same time, we need to pay close attention to the spatio-temporal clustering characteristics of early epidemics and adopt regional epidemic prevention measures.

## Methods

### Data source

The provincial geographic map data of mainland China is obtained from the China Basic Geographic Database (scale 1:1 million), provided by the National Geographic Information Catalog Service Center (http://www.webmap.cn/). The data on the cumulative number of diagnosed cases of COVID-19 is obtained from National Health Commission and the websites of the provincial health commissions, which is obtained through crawler technology provided by python. For some provinces such as Xinjiang and Tibet which are difficult to obtain data on the websites of the health commission, the data is also acquired through web news. The number of new diagnosed cases per day is equal to the difference between the cumulative data of two consecutive days. Since February 12, the national diagnostic criteria for diagnosed cases changed, and “clinical diagnosis” was added to case diagnosis classification in Hubei, the most severely affected province^19^, resulting in inconsistent statistics data caliber. In addition, the onset of cases with a history of contact in Hubei was almost all within 20 days after leaving Hubei, so the research time range of this article is from January 23 to February 11.

Inverse distance weighted interpolation is implemented by arcgis 10.2, Spatio-temporal autocorrelation analysis is done by GeoDa 1.12.1, Spatio-temporal scanning statistics is done by SaTScan 9.5, Time-series diagram of spatio-temporal Moran’s I index changes is drawn by excel 2013, other maps are drawn by arcgis 10.2.

### Model

#### Inverse distance weighted interpolation

The spatial interpolation method is a statistical method that uses known sample data points to estimate unknown data, which can more comprehensively reflect the spatial distribution characteristics of the data. The inverse distance weighted interpolation is the most commonly used. It uses the inverse proportion of the spatial distance between the estimated point and the known data point as the weight for interpolation. The larger the distance, the smaller the weight. The calculation formula is^20^ :

1 $$\begin{aligned} Z=\frac{(\sum _{i=1}^n{\frac{Z_i}{d_i^m}})}{(\sum _{i=1}^n{\frac{1}{d_i^m}})}. \end{aligned}$$

In Eq. (1): Z is the value of the point to be estimated, which is unknown; $Z_i$ is the value of the i-th known sample point around Z; $d_i$ is the distance between Z and $Z_i$; n is the number of known sample points around Z; m is the power value of the inverse distance. The greater the parameter m, the more the point to be estimated will be affected by nearest sample point, and the rougher the space will be. On the contrary, the more distant sample point will be affected and the space will be smoother. Generally, m=2 by default.

#### Spatio-temporal autocorrelation analysis

Spatio-temporal autocorrelation analysis is a special case of bivariate spatial autocorrelation analysis^21^. Spatio-temporal autocorrelation analysis introduces the time dimension on the basis of traditional spatial autocorrelation analysis, which can systematically reflect the spatio-temporal change characteristics and aggregation trends of variables. The spatio-temporal Moran’s I index is a measure of spatio-temporal autocorrelation analysis^22^. The global spatio-temporal Moran’s I index represents the influence of the change of variable i at time t-k on the surrounding variables at time t (k is the lag order). The calculation formula is:

2 $$\begin{aligned} STI_k=\frac{\sum _{i=1}^n\sum _{j=1}^n\omega _{t-k,t}\omega _{ij}(x_{i,t-k}-{\overline{x_{t-k}}})(x_{j,t}-\bar{x_t})}{\sqrt{\sum _{i=1}^n(x_{i,t-k}-{\overline{x_{t-k}}})^2}\sqrt{\sum _{i=1}^n(x_{i,t}-\bar{x_t})^2}}. \end{aligned}$$

In Eq. (2): n is the number of regions; $\omega _{t-k,t}$ is the time weight, which indicates the degree of influence at time t-k on time t, in our results, we always set k=1; $\omega _{ij}$ is the spatial weight; $\ x_{i,t}$ and $\ x_{j,t}$ and $\ x_{i,t-k}$ represent the number of new diagnosed cases in the region at that time, $\overline{x_{t-k}}$ and $\overline{x_{t}}$ represents the average number of new diagnosed cases in all regions at that moment. Due to the lack of epidemic data in Hong Kong, Macao and Taiwan, n=31 in this article. The spatial weight matrix W uses the Queen adjacency type, and the neighboring area of Hainan province is set as Guangdong province. Queen matrix is a kind of spatial weight matrix, which expresses the neighbor relationship between spatial units. If there are n units, queen matrix W can be expressed as follows:

$$\begin{aligned} W= & {} \begin{bmatrix} \omega _{11} &{} \omega _{12} &{} \dots &{} \omega _{1n}\\ \omega _{21} &{} \omega _{22} &{} \dots &{} \omega _{2n}\\ \vdots &{} \vdots &{} \ddots &{} \vdots \\ \omega _{n1} &{} \omega _{n2} &{} \dots &{} \omega _{nn} \end{bmatrix}\\ \omega _{ij}= & {} {\left\{ \begin{array}{ll} 1 &{} if\ i \ is \ adjacent \ to \ j \\ 0 &{} otherwise \end{array}\right. }. \end{aligned}$$

The global spatio-temporal Moran’s I index range is $-\,1 \le \ STK_{i}\le 1$. If it is greater than 0, there is a positive spatio-temporal relationship. If it is less than 0, there is a negative spatio-temporal relationship. If it is equal to 0, there is no spatio-temporal correlation. Under the assumption of normal distribution, the significance test is performed on the null hypothesis that variables do not have spatio-temporal autocorrelation in time and space, that is, they are randomly distributed in time and space. The statistic is the standardized Z value:

3 $$\begin{aligned} Z=\frac{STI_k-E(STI_k)}{\sqrt{Var(STI_k)}}. \end{aligned}$$

The significance level is determined by the p-value calculated by the standardized statistic Z. At the significance level of 0.1, if p < 0.1, it indicates that there is a spatio-temporal aggregation trend. If p > 0.1, it indicates that there is no spatio-temporal aggregation trend, and the variables are randomly distributed in time and space. The global spatio-temporal Moran’s I index is used to detect the overall spatio-temporal correlation. The local aggregation trend and non-stationary information are described by the local spatio-temporal Moran’s I index. The calculation formula is:

4 $$\begin{aligned} PSTI_{i,k}=\frac{n\omega _{t-k,t}(x_{i,t-k}-{\overline{x_{t-k}}})\sum _{j=1}^n\omega _{ij}(x_{j,t}-\bar{x_t})}{\sqrt{\sum _{i=1}^n(x_{i,t-k}-{\overline{x_{t-k}}})^2}\sqrt{\sum _{i=1}^n(x_{i,t}-\bar{x_t})^2}}. \end{aligned}$$

The local spatio-temporal Moran’s I index represents the influence of the number of new diagnosed cases in a local area at time t-k on the number of new diagnosed cases in the surrounding area at time t. Its variables, symbol definitions, value ranges and correlation explanations correspond to the global spatio-temporal Moran’s I index, and the hypothesis test method is also consistent with the global spatio-temporal Moran’s I index test method.

#### Spatio-temporal scanning statistics

The spatio-temporal scanning statistical method is uesd to analyze the data aggregation state based on the moving scanning window of a cylinder^23^. Its circular window is used to scan the spatial area, and the height reflects the time weight information. The cylindrical window moves on the spatio-temporal coordinate system, scanning each spatio-temporal area, and reflecting the state of spatio-temporal aggregation through overlapping cylinders of different sizes and shapes in the spatio-temporal area. This article uses the poisson model’s spatio-temporal scanning statistics to detect the spatio-temporal aggregation area of the daily new diagnosed cases. The significance level p-value calculated according to the actual statistics is used to determine whether the area is a spatio-temporal clustering area. The log-likelihood ratio (LLR) is the grading basis, and the relative risk(RR) represents the risk of an epidemic in the clustering area relative to the surrounding areas. The calculation formula is:

5 $$\begin{aligned} LLR=log\bigg(\frac{c}{\mu }\bigg)\bigg(\frac{C-c}{C-\mu }\bigg)^{(C-c)}. \end{aligned}$$

In Eq. (5), C is the total number of cases in each region, and c is the number of cases in the scanning window. $\mu$ is the expected value of the number of cases in the scanning window. Hypothesis testing is performed on the LLR, and the p-value is calculated by the Monte Carlo method. When p < 0.05, it is considered that there is a significant difference in the RR inside and outside the scan window. The value of LLR corresponds to the possibility of spatio-temporal aggregation in the scanning window, and the scanning window with the largest LLR value corresponds to the most likely aggregation area.

### Ethics declarations

This study used public data from the official website of the National Health Commission of China, all experiments were performed in accordance with relevant guidelines and regulations, and all participants provided written informed consent.

## Data Availability

The COVID-19 monitoring data generated in the current research process can be obtained on the official website of the National Health Commission of China (http://www.nhc.gov.cn/). In the early COVID-19, the public did not know much about the symptoms of this epidemic, some cases may be misdiagnosed as influenza, and some cases were not detected, so there may be some discrepancy between the number of reported cases and the actual number of cases. The software used in this article is ArcGIS 10.2 version (https://developers.arcgis.com/), GeoDa 1.12.1 version (https://spatial.uchicago.edu/), SaTScan 9.5 version (https://www.satscan.org/) statistical computing language and environment.
